# P2X4 receptor stimulation enhances MrgprB2-mediated mast cell activation and pseudoallergic reactions in mice

**DOI:** 10.1038/s41598-022-21667-6

**Published:** 2022-11-03

**Authors:** Kazuki Yoshida, Shota Tanihara, Yuki Miyashita, Kosuke Obayashi, Masa-aki Ito, Kimiko Yamamoto, Toshiyashu Imai, Isao Matsuoka

**Affiliations:** 1 Laboratory of Pharmacology, Faculty of Pharmacy, Takasaki University of Health and Welfare, Takasaki-shi, Gunma, 370-0033 Japan; 2 Department of Biomedical Engineering, Graduate School of Medicine, The University of Tokyo, Tokyo, 113-0033 Japan; 3 Discovery Research Laboratories, Nippon Chemiphar Co., Ltd., Misato, Saitama 341-0005 Japan

**Keywords:** Immunology, Inflammation

## Abstract

Pseudoallergies caused by drugs make disease treatment difficult. Mas-relate G protein-coupled receptor X2 (MRGPRX2), which is specifically expressed in mast cells (MCs), has been implicated in pseudoallergies. High concentrations of therapeutic agents are typically required to stimulate MRGPRX2. Although regulatory mechanisms may enhance this response, the factors involved in this regulation are not well-understood. In this study, the effects of extracellular ATP on MC activation induced by MrgprB2, the mouse ortholog of human MRGPRX2, were examined in mouse peritoneal MCs (PMCs). ATP alone induced minimal PMC degranulation but markedly enhanced degranulation induced by the MrgprB2 agonist compound 48/80 (CP48/80), substance P, PAMP-12, and vancomycin. ATP promoted CP48/80-induced increase in intracellular Ca^2+^ in PMCs. This enhancement effect of ATP was absent in PMCs prepared from P2X4 receptor (P2X4R)-deficient mice and inhibited by the PI3K inhibitor wortmannin. In addition, P2X4R deficiency reduced the skin-specific and systemic anaphylactic responses to CP48/80 in vivo. In MC-deficient *Kit*^W-sh/W-sh^ mice, reconstitution with MCs obtained from wild-type mice led to a more severe anaphylactic response to CP48/80 compared to that from P2X4R-deficient mice. P2X4R-mediated effect may be involved in MrgprB2-mediated MC activation in vivo and is a potential target for alleviating pseudoallergic reactions.

## Introduction

Although pharmacotherapy is an important strategy for treating diseases, adverse reactions to drugs often lead to the discontinuation or modification of drug regimens^1^. The major acute adverse reaction to therapeutic drugs is hypersensitivity^2^, which includes antibody-dependent allergies and antibody-independent pseudoallergic reactions. Antibody-dependent allergies are caused by drug-specific activation of IgE-mediated mast cells (MCs). Pseudoallergies are thought to be mediated by Mas-related G protein-coupled receptor (MRGPR) X2, which recognizes a broad range of therapeutic drugs containing cationic groups, triggering MC activation^3^.

MRGPR is classified into nine major families: MRGPRA–MRGPRH and MRGPRX. MRGPRX receptors are encoded by four different genes that are expressed in sensory nerves^4^. These receptors regulate nociceptor function. Among them, MRGPRX2 (the mouse ortholog is MrgprB2) is highly expressed in connective-tissue MCs, including peritoneal-cavity and skin-resident MCs, and it mediates MC activation via various basic secretagogues, such as compound 48/80 (CP48/80), substance P, and PAMP-12^5,6^. In addition, the MRGPRX2 mediates pseudoallergic reactions induced by various clinically important drugs, including morphine, codeine, tubocurarine, and ciprofloxacin^4^. High concentrations of these compounds are typically required to activate MC degranulation; a recent study showed that mild-to-moderate allergic-type acute adverse events to therapeutic drugs that can stimulate MRGPRX2 are much more common than previously considered^2^. Therefore, the responsiveness of MCs to MRGPRX2-mediated signaling may be upregulated under certain conditions; however, the underlying mechanism remains unclear.

MC activation is regulated by signaling through various receptors. The IgE-FcεRI-mediated degranulation response to antigens is promoted by Gi-coupled receptor stimulation and is suppressed by Gs-coupled receptor stimulation and inhibitory receptors with immunoreceptor tyrosine-based inhibition motif^7,8^. We previously reported that extracellular ATP also enhances MC degranulation^9,10^ via the P2X4 receptor (P2X4R). P2X4R is a ligand-gated nonselective cation channel with high Ca^2+^ permeability^11^. Stimulation of P2X4R enhances both antigen-dependent and antigen-independent degranulation, such as via prostaglandin E_2_ (PGE_2_)- and adenosine-mediated effects. P2X4R-mediated upregulation of antigen-induced degranulation is accompanied by increased phosphorylation of tyrosine kinase Syk, whereas that of antigen-independent degranulation induced by co-stimulation with PGE_2_ or adenosine is mediated by Gi-dependent signaling^9^. Because MRGPRX2 is a Gi-coupled receptor, extracellular ATP may affect the pseudoallergic response mediated by MRGPRX2 activation.

In this study, we investigated the effects of extracellular ATP on MC degranulation induced by the activation of MrgprB2, the mouse homologue of human MRGPRX2. In our previous study, we observed ATP-induced upregulation of antigen-dependent and antigen-independent degranulation in bone marrow-derived MCs (BMMCs)^9,10^. However, BMMCs present with an immature phenotype and show muted response to MgprB2 agonists; therefore, we used mouse peritoneal MCs (PMCs), which respond well to MrgprB2 stimulation.

## Results

### P2 receptor expression and their role in the degranulation response in PMCs

We first analyzed P2 receptor expression in PMCs using quantitative reverse transcription polymerase chain reaction (PCR). The PMCs expressed ionotropic P2X1, X4, and X7 and the G protein coupled P2Y_1_ and P2Y_14_ receptors (Supplementary Fig. S1a,b). These expression profiles and levels were similar to those in BMMCs. Functional expression of these P2 receptors was evaluated by measuring changes in intracellular Ca^2+^ concentrations ([Ca^2+^]i) (Supplementary Fig. S1d). Low concentrations of ATP (100 μM), which activate P2X1 and P2X4R, the P2X1 agonist α,β-methylen ATP, P2Y_1_ agonist ADP, and P2Y_14_ agonist UDP-glucose (UDP-G), induced increases in [Ca^2+^]i. High concentrations of ATP (1 mM) led to a large and sustained increase in [Ca^2+^]i, a typical response mediated by the P2X7 receptor. Using the receptor agonists, we next examined the effects of P2 receptor stimulation on PMC degranulation. In contrast to the Ca^2+^ responses, different P2 receptor agonists induced minimal degranulation in PMCs, except for BzATP, a potent P2X7 agonist (Supplementary Fig. S1e,f). 2'(3')-O-(4-benzoylbenzoyl) ATP (BzATP, 300 μM) alone induced marked degranulation; this effect was completely inhibited by the P2X7 receptor antagonist AZ10606120 (10 μM). These results suggest that among the various P2 receptors expressed in PMCs, only the P2X7 receptor can directly induce degranulation in these cells.

### Effects of ATP on CP48/80-induced degranulation in PMCs

**Figure 1:**
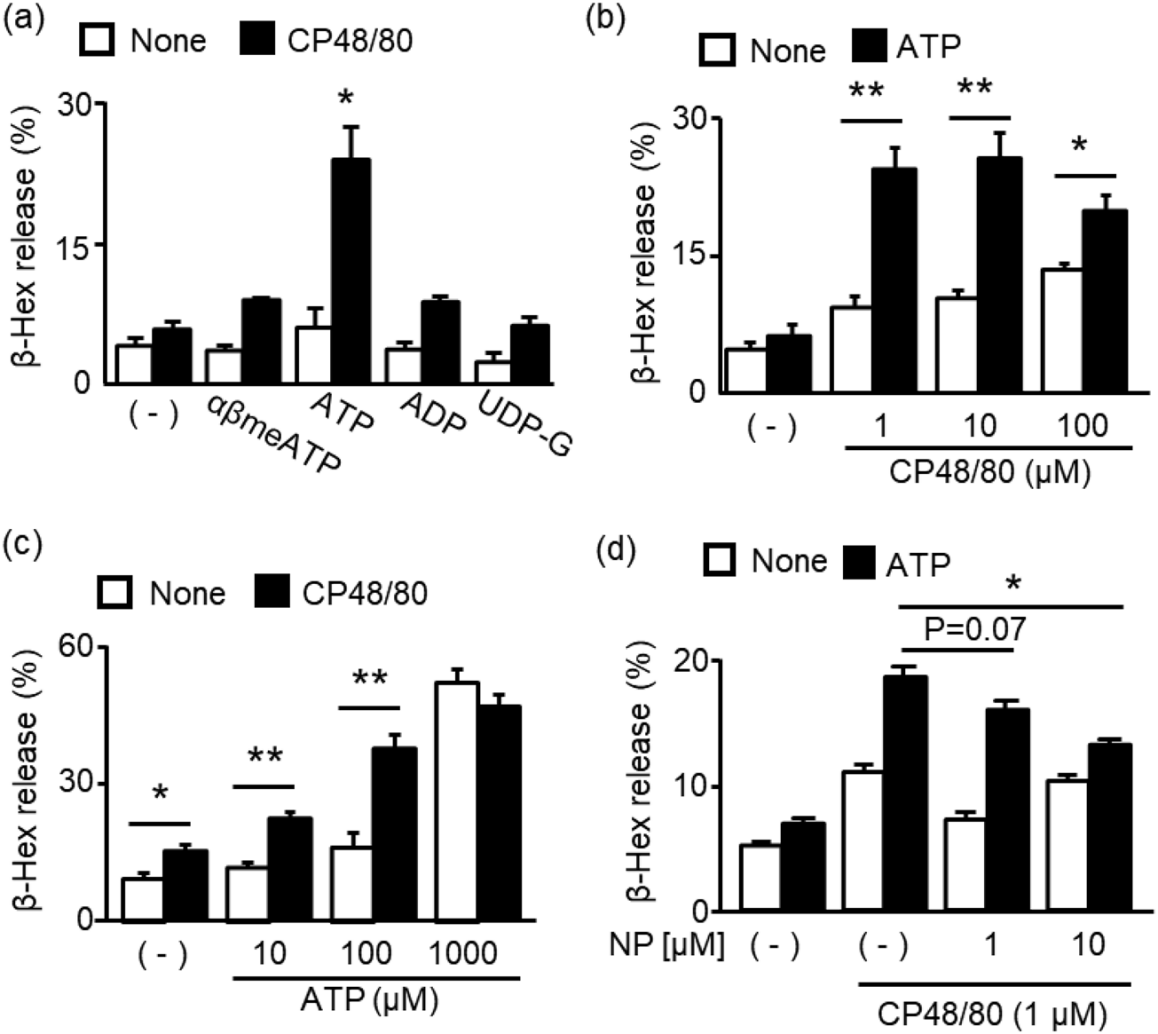
Effects of purinergic receptor agonists on CP48/80-induced degranulation in PMCs. (**a**) PMCs were stimulated with αβmeATP (10 μM), ATP (100 μM), ADP (100 μM), or UDP-G (100 μM) in the presence or absence of CP48/80 (1 μM) for 10 min. (**b**) PMCs were stimulated with CP48/80 (1–100 μM) in the presence or absence of ATP (100 μM) for 10 min. (**c**) PMCs were stimulated with ATP (10–1000 μM) in the presence or absence of CP48/80 (1 μM). (**d**) PMCs were stimulated with ATP (100 μM) and CP48/80 (1 μM) in the presence or absence of P2X4R antagonist NP-1815-PX (NP, 1 or 10 μM). Data are presented as the mean ± SEM (n = 3). *P < 0.05, **P < 0.01.

We previously reported that lower concentrations of ATP that are not sufficient to stimulate the P2X7 receptor enhance IgE-dependent and -independent degranulation in BMMCs^9,10^. Therefore, we examined the effects of nucleotide agonists such as αβmeATP, ATP, ADP, or UDP-G on MrgprB2-mediated degranulation using 1 μM MrgprB2 agonist CP48/80 at the concentration, which induced week degranulation in PMCs. Among the different agonists tested, only ATP enhanced CP48/80-induced degranulation in PMCs (Fig. 1a). We performed more detailed analysis of the synergies between ATP and CP48/80 on degranulation in PMCs. Stimulation of MrgprB2 with CP48/80 induced degranulation in a concentration-dependent manner. In the presence of ATP (100 μM), CP48/80-induced degranulation was markedly enhanced with an increase in the maximal degranulation response (Fig. 1b). In contrast, ATP at concentration of up to 100 μM did not induce substantial degranulation, whereas a marked response was induced at 1 mM. In the presence of CP48/80 (1 μM), lower concentrations of ATP induced a concentration-dependent degranulation reaction, whereas the reaction at 1 mM remained unaffected (Fig. 1c). Furthermore, ATP-induced enhancement of degranulation by CP48/80 was inhibited by the P2X4R antagonist NP-1815-PX in a concentration-dependent manner (Fig. 1d). These results suggest that ATP enhanced CP48/80-induced degranulation via P2X4R but not via other P2 receptors including P2X7 receptors.

### Effects of P2X4R stimulation on degranulation induced by MrgprB2 agonists

**Figure 2:**
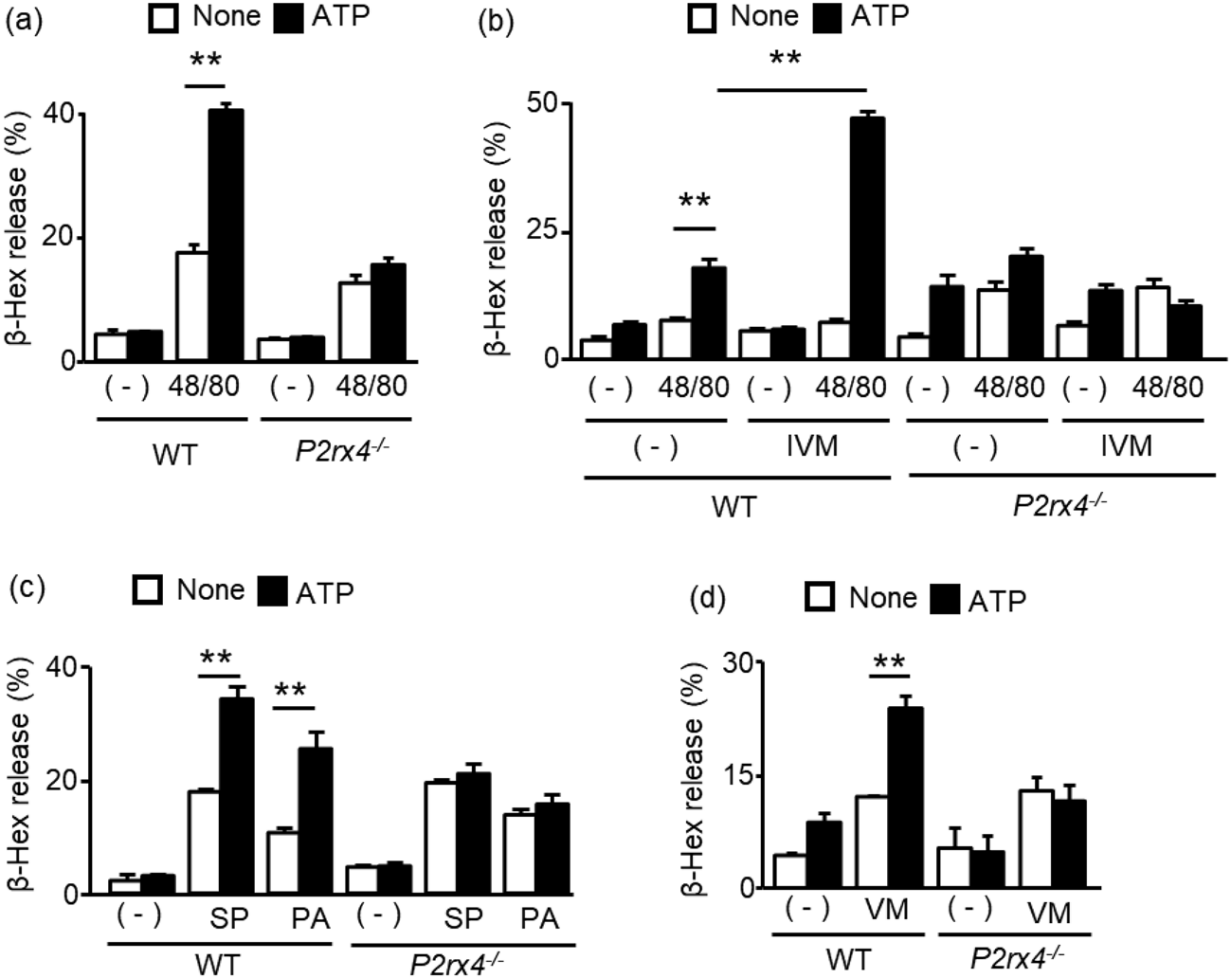
Role of P2X4R in ATP-induced enhancement of degranulation responses to MrgprB2 agonists. (**a**) WT or *P2rx4*^*−/−*^ PMCs were stimulated with CP48/80 (48/80, 1 μM) in the presence or absence of ATP (100 μM). (**b**) WT or *P2rx4*^*−/−*^ PMCs were stimulated with 48/80 (1 μM) and ATP (100 μM) in the presence or absence of ivermectin (IVM, 1 μg/ml). (**c** and **d**) WT or *P2rx4*^*−/−*^ PMCs were stimulated with Substance P (SP, 100 μM), proadrenomedullin N-terminal 20 peptide (PA, 10 μM), and vancomycin (VM, 1 mM) in the presence or absence of ATP (100 μM). Data are presented as the mean ± SEM (n = 3). **P < 0.01.

To examine whether P2X4R is involved in the synergistic degranulation response induced by ATP and CP48/80, we compared the responses of PMCs obtained from wild-type (WT) and *P2rx4* deficient (*P2rx4*^*−/−*^) mice. P2 receptors as well as MrgprB2 expression in *P2rx4*^*−/−*^ PMCs did not significantly differ from that in PMCs obtained from WT mice except for the absence of P2X4R (Supplementary Fig. S1a–c). As shown in Fig. 2a, CP48/80-induced degranulation in *P2rx4*^*−/−*^ PMCs was not enhanced by ATP. Moreover, the P2X4R positive allosteric modulator ivermectin promoted the effect of ATP in WT PMCs but not in *P2rx4*^*−/−*^ PMCs (Fig. 2b). MrgprB2 recognizes various cationic molecules including neuropeptide and therapeutic medicines. Substance P (SP) and proadrenomedullin N-terminal 20 peptide (PA) stimulate MrgprB2, causing MC degranulation and subsequent itching, pain, and skin inflammation^12,13^. In addition, vancomycin (VM), an important antibiotic against methicillin-resistant *Staphylococcus aureus*, stimulates MC MrgprB2 to induce redneck syndrome^14^. We found that stimulation of PMCs with SP, PA and vancomycin induced degranulation. ATP enhanced these agonist-induced degranulation in a P2X4R expression-dependent manner; these effects were not observed in *P2rx4*^*−/−*^ PMCs (Fig. 2c,d).

### Mechanism underlying the synergistic effect of P2X4R and MrgprB2 stimulation

**Figure 3:**
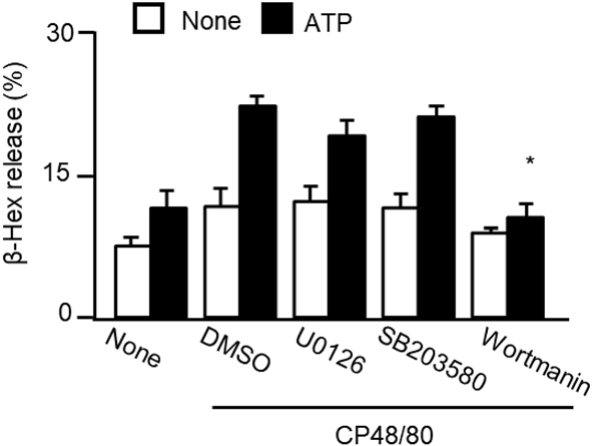
Effects of kinase inhibitors on MC degranulation induced by co-stimulation with ATP and CP48/80. WT PMCs were stimulated with ATP and CP48/80 in the presence or absence of the MEK1/2 inhibitor U0126 (5 μM), p38 MAP kinase inhibitor SB203580 (10 μM), and PI3K inhibitor wortmannin (0.1 μM). The results are presented as the mean ± SEM (n = 3). *P < 0.05 vs DMSO treated ATP plus CP48/80.

MC degranulation is regulated by various protein kinases, such as the extracellular signal regulated kinase 1/2, p38 mitogen-activated protein kinase (MAPK), and phosphoinositide 3-kinase (PI3K) signaling pathways. We investigated the effects of the MEK1/2 inhibitor U0126, p38 MAPK inhibitor SB203580, and PI3K inhibitor wortmannin on degranulation induced by co-stimulation with ATP and CP48/80. ATP-enhanced degranulation was inhibited by wortmannin but not by SB203580 (Fig. 3). U0126 tended to inhibit the increased degranulation caused by ATP and CP48/80, but this effect was not significant.

**Figure 4:**
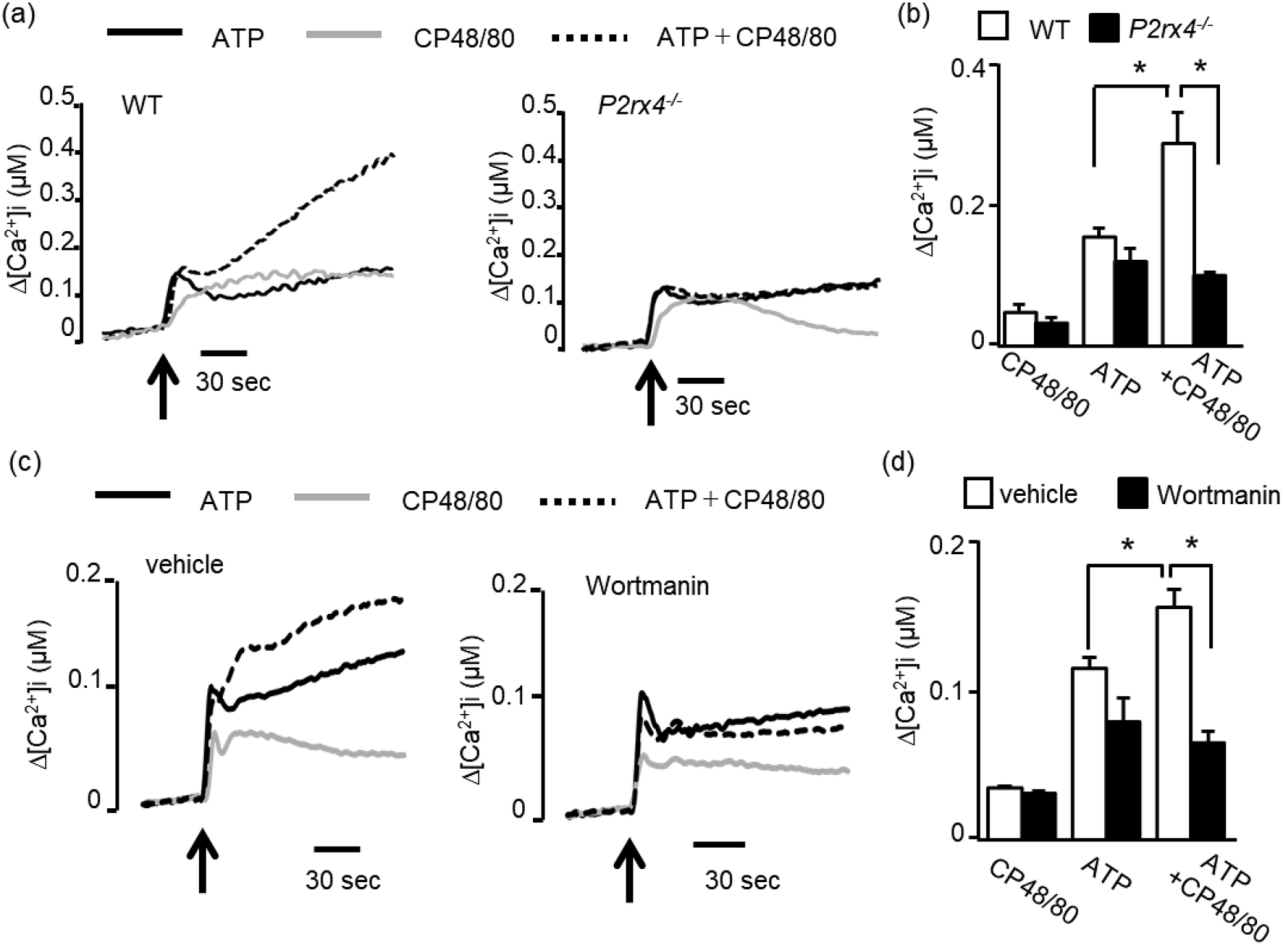
Effect of co-stimulation with ATP and CP48/80 on intracellular Ca^2+^ concentration ([Ca^2+^]i) levels in PMCs. (**a**) PMCs prepared from WT (left) or *P2rx4*^*−/−*^ (right) mice were loaded with Fura-2 AM, and changes in [Ca^2+^]i were monitored after stimulation with ATP (black line, 100 μM), CP48/80 (gray line, 1 μM), or ATP plus CP48/80 (dotted line) at the time indicated by the arrow. The Ca^2+^ data are representative of 4–5 independent experiments. (**b**) Summary of the data obtained in **a**. Results are shown based on [Ca^2+^]i at 120 s after stimulation (WT, n = 4; *P2rx4*^*−/−*^, n = 5). (**c**) WT PMCs were loaded with Fura-2 AM and preincubated for 2 min with vehicle or wortmannin (0.1 μM). Changes in [Ca^2+^]i were monitored after stimulation with ATP (black line, 100 μM), CP48/80 (gray line, 1 μM), or ATP plus CP48/80 (dotted line) at the time indicated by the arrow. The Ca^2+^ data are representative of 4–5 independent experiments. (**d**) Summary of the data obtained in **c**. Results are shown based on [Ca^2+^]i at 120 s after stimulation (n = 4). Data are shown as the mean ± SEM. *p < 0.05.

We recently showed that P2X4R-mediated Ca^2+^ influx is enhanced by Gi-coupled receptor activation in a PI3K-dependent manner, enhancing the increase in [Ca^2+^]i^9^. Therefore, we investigated the Ca^2+^ responses to ATP and CP48/80. In fura-2-loaded WT*-*PMCs, ATP caused a transient increase in [Ca^2+^]i followed by a sustained increase, whereas CP48/80 induced only a weak increase in [Ca^2+^]i. Co-stimulation with ATP and CP48/80 resulted in a marked increase in the sustained elevation of [Ca^2+^]i without affecting the initial transient Ca^2+^ (Fig. 4a). In *P2rx4*^*−/−*^ PMCs, the Ca^2+^ response to ATP or CP48/80 did not differ from that in WT PMCs. However, the sustained [Ca^2+^]i elevation induced by co-stimulation with ATP and CP48/80 in WT PMCs was absent in *P2rx4*^*−/−*^ PMCs (Fig. 4a,b). In addition, the increase in [Ca^2+^]i induced by co-stimulation with ATP and CP48/80 was inhibited by the PI3K inhibitor wortmannin (Fig. 4c,d).

### Role of P2X4R signal in CP48/80-induced pseudoallergic reactions

**Figure 5:**
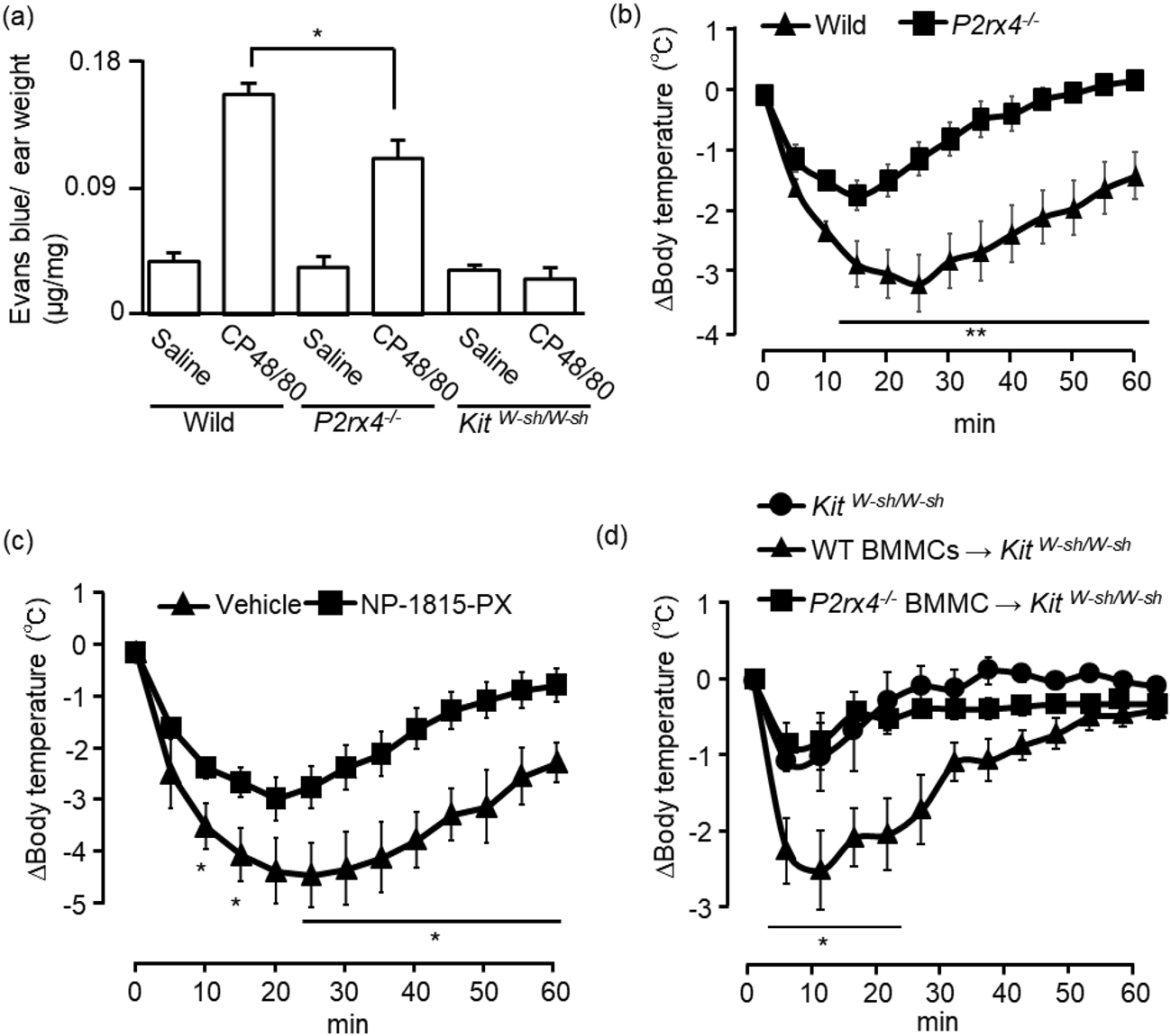
Role of P2X4R on pseudoallergic response induced by CP48/80 in mice. (**a**) WT, *P2rx4*^*−/−*^, and *Kit*^*W-sh/W-sh*^ mice were injected intravenously with Evans blue, and then the mouse ear was intradermally injected with saline or CP48/80 (100 ng/20 μL), and extravasated Evans blue dye was measured (n = 5). (**b**) WT or *P2rx4*^*−/−*^ mice were injected intravenously with CP48/80 (50 μg/100 μL), and rectal temperatures were measured every 5 min for 60 min (WT n = 8, *P2rx4*^*−/−*^ n = 7). (**c**) NP-1815-PX (10 mg/kg) intraperitoneally injected mice were injected intravenously with CP48/80 (50 μg/100 μL), and rectal temperatures were measured every 5 min for 60 min (saline n = 4, NP-1815-PX n = 8) (**d**) *Kit*^*W-sh/W-sh*^ mice were reconstituted with WT (WT BMMCs → *Kit*^*W-sh/W-sh*^; n = 4) or *P2rx4*^*−/−*^ (*P2rx4*^*−/−*^ BMMC → *Kit*^*W-sh/W-sh*^; n = 3) BMMCs. Reconstituted mice were injected intravenously with CP48/80 (50 μg/100 μL), and rectal temperatures were measured every 5 min for 60 min. Data are presented as the mean ± SEM. *P < 0.05, **P < 0.01.

Administration of CP48/80 to mice triggers pseudoallergic reactions via MrgprB2-mediated MC activation^5^. Therefore, we next investigated whether P2X4R-stimulated effect contributes to pseudoallergic reactions induced by CP48/80 in vivo. Intradermal injection of CP48/80 into the auricle of WT mice caused significant Evans blue extravasation compared to that in the saline-injected auricles (Fig. 5a). This response was absent in MC-deficient *Kit*^W-sh/W-sh^ mice (Fig. 5a), suggesting a MCs-dependent response. CP48/80-induced Evans blue extravasation was significantly lower in *P2rx4*^*−/−*^ mice (Fig. 5a). In WT mice, intravenous injection of CP48/80 induced a rapid decrease in the rectal temperature, which slowly recovered, whereas *P2rx4*^*−*/*−*^ mice showed a much smaller decrease in the rectal temperature, which recovered quickly (Fig. 5b). We further investigated effect of the selective P2X4R antagonist NP-1815-PX^15^ on the CP48/80-induced pseudoallergic reaction. Pretreatment of WT mice with NP-1815-PX significantly alleviated CP48/80-induced reductions in the rectal temperature (Fig. 5c). We finally examined whether the mild pseudoallergic response to CP48/80 in *P2rx4*^*−/−*^ mice resulted from decreased MC responses because of MC P2X4R deficiency in vivo. MC-deficient *Kit*^W-sh/W-sh^ mice were reconstituted either with BMMCs prepared from WT mice or those from *P2rx4*^*−/−*^ mice. The reconstruction of mast cells in *Kit*^W-sh/W-sh^ mice was confirmed by staining the lung sections with avidin-rhodamine and toluidine blue (Supplementary Fig. S2). The MC distribution was similar between *Kit*^W-sh/W-sh^ mice reconstituted with WT and *P2rx4*^*−/−*^ BMMCs. Intravenous administration of CP48/80 rapidly decreased the rectal temperature of WT BMMCs-reconstituted mice, whereas this decrease was quite mild in *P2rx4*^*−/−*^ BMMC-reconstituted mice (Fig. 5d).

## Discussion

We demonstrated that extracellular ATP enhanced the degranulation reaction following different MrgprB2 stimuli in mouse ice PMCs. PMCs express various ATP receptors including the ionotropic P2X1, P2X4, and P2X7 receptors and G protein-coupled P2Y_1_ and P2Y_14_ receptors. Functional expression of these receptor subtypes was confirmed by measuring [Ca^2+^]i with an agonist that selectively stimulated each receptor subtype. Our results indicate that enhancement of the CP48/80-mediated degranulation reaction by ATP was mediated by the P2X4R. First, although ATP acts as an agonist for all P2X receptors, the concentration used (100 μM) did not activate the P2X7 receptor, as concentrations higher than 500 μM were required. In addition, αβmeATP, a P2X1 receptor agonist, did not induce this effect. ATP may stimulate the P2Y_1_ receptor, but the more potent P2Y_1_ receptor agonist ADP was ineffective. Involvement of the P2Y_14_ receptor can be excluded based on its lack of sensitivity to ATP and to UDP-G, a specific agonist for this receptor. These pharmacological features suggest the involvement of P2X4R. Indeed, ATP-enhancement of CP48/80-mediated degranulation was absent in PMCs from *P2rx4*^*−/−*^ mice. PMCs prepared from *P2rx4*^*−/−*^ mice showed no changes in the expression of P2 receptors and MrgprB2, except for the deficiency of P2X4R. These results indicate that P2X4Rs specifically enhance MrgprB2-induced degranulation in PMCs.

MCs exhibit diverse phenotype depending on the tissue environment^16^. We previously demonstrated in BMMCs that P2X4R stimulation enhances degranulation induced by antigen-induced IgE-FcεRI activation^17^. Because BMMCs are poorly responsive to MrgprB2 agonists such as CP48/80, we used PMCs that respond to MrgprB2 stimuli. In our previous study of BMMCs, P2X4R stimulation did not significantly affect the antigen-induced intracellular Ca^2+^ elevation but augmented the antigen-induced tyrosine kinase signaling pathway, including Syk and phospholipase Cγ^10^. The P2X4R signal also enhanced degranulation response to PGE_2_, which stimulates the Gi-coupled EP_3_ receptor, leading to increased P2X4R-mediated Ca^2+^ influx in a manner dependent on phosphatidylinositol-3-kinase activation^9^. Therefore, the P2X4R signal may influence the degranulation reaction induced by activation of the IgE-FcεRI and EP_3_ receptors through different mechanisms. As MrgprB2 is a Gi-coupled receptor, the P2X4R signal enhances the MrgprB2-mediated response though a mechanism similar to its effect on the EP_3_ receptor-mediated response. Indeed, the Ca^2+^ response of PMCs mediated by ATP or CP48/80 alone was markedly increased by their co-stimulation. This co-stimulation-induced enhancement in the Ca^2+^ response was not observed in *P2rx4*^*−/−*^ PMCs. MrgprB2-induced [Ca^2 +^]i elevation is due to activation of PLCβ by Gβγ, followed by storage-operated Ca^2+^ entry mediated by STIM1/2 and the Orai channel. However, a previous study did not support that P2X4R stimulation facilitates such MrgprB-induced Ca^2+^ signaling. The function of P2X4R is upregulated by PI3K-mediated accumulation of phosphatidylinositol-3,4,5-trisphosphate in the cell membrane to increase Ca^2+^ influx^18^. In line with this, the enhanced Ca^2+^ response and degranulation induced by co-stimulation with P2X4R and MrgprB2 were inhibited by the PI3K inhibitor wortmannin. Although stimulation of MrgprB2 in MCs activates PI3K^19^, as we previously showed, stimulation of P2X4R in MCs has little effect on the PI3K signaling pathway^9,10^. These results suggest that wortmannin inhibits degranulation and the Ca^2+^ response by inhibiting MrgprB2-induced PI3K activation.

Our in vivo experiments using WT and *P2rx4*^*−/−*^ mice showed that CP48/80-induced auricular vascular hyper permeability was significantly reduced in *P2rx4*^*−/−*^ mice. Furthermore, the decrease in the rectal temperature due to the CP48/80-induced systemic pseudoallergic reaction was reduced in *P2rx4*^*−/−*^ mice. We previously reported that the passive anaphylactic response was significantly mild in IgE-sensitized *P2rx4*^*−/−*^ mice. In addition, there is no difference in the decrease in the rectal temperature when histamine is administered to WT and *P2rx4*^*−/−*^ mice^10^, suggesting that the difference observed in WT and *P2rx4*^*−/−*^ mice was not due to the difference in the sensitivity to histamine. These results indicate that the responsiveness of MCs in vivo is constitutively upregulated by P2X4R stimulation. This prediction was confirmed by the finding that the decrease in the rectal temperature due to the CP48/80-induced systemic pseudoallergic reaction in WT mice was alleviated by the P2X4R antagonist NP-1850-NP. Furthermore, experiments in MC-deficient *Kit*^*W-sh/W-sh*^ mice, in which MCs were reconstituted with WT BMMCs or *P2rx4*^*−/−*^ BMMCs, revealed that the reduced CP48/80-induced systemic pseudoallergic reaction occurred because of the deficiency of MC P2X4R. In this model, BMMCs used for reconstruction into MC-deficient *Kit*^*W-sh/W-sh*^ mice are known to maturate in migrating tissues and acquire responsiveness to CP48/80^20^. Although more detailed analysis, such as changes in serum histamine levels in response to CP48/80 stimulation, is required, our results suggest that P2X4R in MCs plays an important role in CP48/80 responsiveness in different allergic models in mice.

ATP is released from intracellular-enriched sources through various regulated mechanisms or via passive leakage because of cellular damages and acts as an intercellular mediator^21^. In the skin, ATP is released from keratinocytes by physical irritation and inflammation^22^. Scratching behavior against itching caused by irritants and allergic reactions induced mechanical stimulation-induced ATP release, which further enhanced MrgprB2-induced MC activation via P2X4R. In addition, ATP is packed in the neurotransmitter vesicle and co-release with neuropeptide that stimulates MrgprB2. In this study, the P2X4R signal promoted degranulation not only via CP48/80, but also through peptide ligands such as substance P and PAMP-12. Because MCs often exist near nerve endings, effects induced by co-stimulation of P2X4R and MrgprB2 may be important for understanding neuropeptide-induced MC-dependent responses^12^. In addition to the immediate degranulation reaction, the MrgprB2 signal is well-known to be involved in pseudoallergic inflammation by promoting the production of inflammatory cytokines in MCs^13^. We previously showed that co-stimulation of P2X4R and the prostanoid EP_3_ receptor promotes not only MC degranulation, but also cytokine production^9,23^. Therefore, it is important to investigate whether P2X4R stimulation affects MrgprB2-mediated cytokine production in MCs.

MrgprB2 recognizes cationic drugs and mediates pseudoallergic reactions^2^. For example, peptide drugs, neuromuscular blocking agents, morphine, and antibacterial drugs such as fluoroquinolones and vancomycin stimulate MrgprB2 to induce MC degranulation^24,25^. We showed that P2X4R stimulation also enhances vancomycin-induced PMC degranulation. In contrast, some drug-induced allergic reactions, such as those caused by β-lactam antibiotics, are mediated by IgE-dependent mechanisms that are also enhanced by P2X4R stimulation. Therefore, it may be necessary to consider the role of P2X4R-stimulated effect to understand drug-induced allergic reactions.

In conclusion, extracellular ATP stimulates P2X4R, resulting in enhancement of MrgprB2-mediated PMC degranulation and exacerbating the MrgprB2-induced pseudoallergic reaction in vivo. MrgprB2-mediated MC activation has been implicated in various disease models, such as allergic contact dermatitis, irritant contact dermatitis, rosacea, atopic dermatitis, and skin infection^26^; therefore, MrgprB2 antagonists have received attention as therapeutic agents to treat these diseases^27^. Our results suggest that P2X4R-stimulated effect is also an important target with therapeutic potential for MC-dependent inflammation.

## Methods

### Materials

ATP, ADP, adenosine, αβmeATP, BzATP, UTP, UDP, UDP-G, 2,4-DNP human serum albumin (DNP-HSA), anti-DNP IgE (clone SPE-7), CP48/80, *p*-nitrophenyl *N*-acetyl-b-d-glucosaminide, fura-2-acetoxymethylester (fura-2AM), and ivermectin were from Sigma-Aldrich (St. Louis, MO, USA). Substance P and proadrenomedullin N-terminal 20 peptide were from Peptide Institute (Osaka, Japan). Allophycocyanin-conjugated rat anti-mouse CD117 (c-Kit) Ab (clone 2B8) was from BD Pharmingen (Franklin Lakes, NJ, USA). PE-conjugated mouse anti-mouse FcεRIα Ab (clone MAR-1) was from eBioscience (San Diego, CA). Recombinant mouse IL-3 and recombinant mouse stem cell factor were from PeproTech (Rocky Hill, NJ, USA). NP-1815-PX was provided by Nippon Chemiphar Co., Ltd. (Tokyo, Japan). SB203580 (p38 MAPK inhibitor) and wortmannin (PI3K inhibitor) were from Cayman Chemical (Ann Arbor, MI, USA). U0126 (MEK1/2 inhibitor) was from Cell Signaling Technology (Danvers, MA, USA). All other chemicals used were of reagent grade or of the highest quality available.

### Animals

All animal experiment protocols were approved by the Animal Research Committee of Takasaki University of Health and Welfare (approval number No. 2033), and conducted according to the Animal Experiment Regulations of Takasaki University of Health and Welfare. The study was carried out in compliance with the Animal Research: Reporting of In Vivo Experiments (ARRIVE) guidelines. C57BL/6 mice, 7–10 weeks old, were obtained from SLC Japan (Hamamatsu, Japan). MC deficient *Kit*^W-sh/W-sh^ mice (RBRC01888) were provided by RIKEN BRC through the National BioResource Project of the MEXT/AMED (Tsukuba, Japan). *P2rx4*^*−/−*^ mice were prepared on a C57BL/6 background ^28^. Mice were maintained under specific pathogen-free conditions with a 12-h light–dark cycle and free access to feed and water at room temperature of 22 ± 2 °C.

### Mast cell preparation and culture

Bone marrow-derived MCs (BMMCs) were prepared from bone marrow cells obtained from C57BL/6 wild-type WT and *P2rx4*^*−/−*^ mice^10^. Briefly, to prepare the BMMCs, bone marrow cells were cultured in RPMI1640 growth medium containing 10% fetal bovine serum, 100 U/mL penicillin, 100 mg/mL streptomycin, and 10 ng/mL recombinant IL-3. After 2 weeks, the cells were cultured in the presence of 10 ng/mL recombinant stem cell factor for 4–6 weeks, when more than 95% of cells were double-positive for c-Kit and FcεRI, as shown in a previous study^17^. PMCs were prepared from peritoneal cells obtained from WT and *P2rx4*^*−/−*^ mice^10^. Briefly, mouse peritoneal cells were collected by washing the peritoneal cavity with 4 mL RPMI1640 medium and then cultured in RPMI1640 medium containing 10% fetal bovine serum, 100 U/mL penicillin, 100 μg/mL streptomycin, 10 ng/mL recombinant IL-3, and 10 ng/mL recombinant stem cell factor for 14 days, after which more than 95% of cells were double-positive for c-Kit and FcεRI.

### Quantitative reverse transcription-PCR

Total RNA was isolated using a NucleoSpin RNA kit (Macherey–Nagel, Düren, Germany). First-strand cDNA was synthesized using Moloney-murine leukemia virus reverse transcriptase with 6-mer random primers (Takara Bio, Shiga, Japan), and quantitative reverse transcription-PCR was performed using TB Green Premix Ex TaqII (Tli RNaseH Plus) (Takara Bio). The results were normalized to the expression level of glyceraldehyde-3-phosphate dehydrogenase (*GAPDH*). The primer sequences used in real-time PCR were shown in Table 1.

**Table 1:** Primer sequences.

	Forward	Reverse
*P2X1*	gaaggtggcatatgccaaga	tgtcatccacctctacagga
*P2X2*	acttcgtgtggtacgtcttc	tgatccccttgactttggtg
*P2X3*	ggattctgtccagagaatgagg	ggcttccatcatgataggca
*P2X4*	aacatgatcgtcaccgtgaa	aatggaacacaccttccagt
*P2X5*	gaaaactggtcgctgtctac	gtagagattggtggagctga
*P2X6*	ttgcaacctggacacgaa	tttctgctgcagctggaa
*P2X7*	taagctgtaccagcggaaag	tgcaaagggaaggtgtagtc
*P2Y1*	cagaccccagaaatgtgtga	ctcgtgtctccattctgctt
*P2Y2*	gcctgtgcatatgtgagtgaag	cacgtacttgaagtcctcgttg
*P2Y4*	gtcttctctgcctaggtgtt	ccagagtgatcaagaaggga
*P2Y6*	tgcctttccacatcacca	gagcctctgtaagagatcgt
*P2Y12*	tcagccaataccaccttctc	tcctcattgccaagctgt
*P2Y13*	ccatgtgtgagatggggaaa	gctagggtgatgttgtctgt
*P2Y14*	tccagccgcaatatcttcag	ataatggggtccagacacac
*MrgprB2*	acgtccaagacacacatcag	tagacccacagagaacacca
*GAPDH*	tgctgagtatgtcgtggagt	catacttggcaggtttctcc

### Degranulation assay

Degranulation was evaluated by measuring β-hexosaminidase release^17^. PMCs were sensitized with 50 ng/mL anti-DNP-IgE overnight in RPMI1640 growth medium. The cells were washed twice and suspended in Krebs–Ringer HEPES buffer (KRH; 130 mM NaCl, 4.7 mM KCl, 4.0 mM NaHCO_3_, 1.2 mM KH_2_PO_4_, 1.2 mM MgSO_4_, 1.8 mM CaCl_2_, 11.5 mM glucose, and 10 mM HEPES [pH 7.4]) containing 0.1% bovine serum albumin (BSA). The cells were preincubated at 37 °C for 5 min when using kinase inhibitors or receptor antagonists and stimulated under various conditions at 37℃ for 10 min. The reactions were terminated by placing the samples on ice, followed by centrifugation at 300 × *g* for 5 min. The supernatants were collected, and the cell pellets were then lysed in 0.1% Triton X-100. The supernatant and cell lysates were incubated with an equal volume of 1 mM *p*-nitrophenyl *N*-acetyl-b-d-glucosaminide dissolved in citrate buffer (pH 4.5) in a 96-well plate at 37 °C for 30 min. The reactions were stopped by adding 0.1 M sodium carbonate buffer (pH 10.4), and the absorbance was measured at 405/655 nm. The percentage degranulation was calculated as follows: β-hexosaminidase release (%) = supernatant absorbance/(supernatant absorbance + lysate absorbance) × 100.

### Intracellular Ca^2+^ concentration ([Ca^2+^]i) measurement

Cells were collected and washed twice with KRH containing 0.1% BSA, suspended in KRH-BSA buffer, and loaded with 1 μM Fura-2 AM at 37 °C for 30 min. The Fura-2-loaded cells were washed twice with KRH-BSA buffer. Changes in Fura-2 fluorescence were measured with an F-2700 (Hitachi, Tokyo, Japan). The excitation wavelengths were 340 and 380 nm, and Fura-2 fluorescence emission was measured at 510 nm. After measurement, Triton X-100 was added to the cell suspension to obtain the maximum fluorescence, and then excess EDTA was added to obtain the minimum fluorescence. [Ca^2+^]i was calculated as the ratio of fluorescence at the two excitation wavelengths, with a K_d_ value of 224 nM for Fura-2–Ca^2+^ equilibrium.

### Passive systemic anaphylaxis

WT and *P2rx4*^*−/−*^ mice were intravenously injected with 50 μg of CP48/80 in 100 μL saline. The P2X4R antagonist NP1815-PX (10 mg/kg) was intraperitoneally injected at 15 min before CP48/80 injection. After CP48/80 injection, the rectal temperature was measured every 5 min for 60 min with a digital thermometer (Physitemp Instruments, Clifton, NJ, USA). For MCs reconstitution experiments, WT and *P2rx4*^*−/−*^ BMMCs were cultured; after the cells were mature, 5 × 10^6^ cells were injected into MC-deficient *Kit*^*W-sh/W-sh*^ mice, 4–6 weeks old, via the tail vein. After 16 weeks, the MC-reconstituted *Kit*^W-sh/W-sh^ mice were subjected to CP48/80-induced passive systemic anaphylaxis experiments.

### Passive cutaneous anaphylaxis

Mice were anesthetized with isoflurane, injected intravenously with 200 μL 0.5% Evans blue diluted in PBS, and injected intradermally in the right ear with CP48/80 100 ng/20 μL saline and in the left ear with the vehicle 0.1% dimethyl sulfoxide in saline. After 30 min, the mice were euthanized by cervical dislocation, and their ears collected and weighed. Evans blue dye was extracted from the ears with 1 mL formamide at 55 ℃ for 24 h, and absorbance was measured at 620 nm. Data are expressed as μg of Evans blue per mg of ear.

### Statistics

All experiments were repeated at least three times, yielding similar results. Data represent the mean ± standard error of the mean. Statistical analyses were performed using the Student’s *t*-test for sample comparisons and one-way analysis of variance with Dunnett’s two-tailed test for multiple comparisons. P values < 0.05 were considered to indicate statistically significant results.

## Supplementary Information

Supplementary Figures.

## Data Availability

The datasets generated during and/or analyzed during the current study are available from the corresponding author on reasonable request.
