# Supplementary material for: P2X4 receptor stimulation enhances MrgprB2-mediated mast cell activation and pseudoallergic reactions in mice
Source: Sci Rep. 2022 Nov 3;12:18613. doi: 10.1038/s41598-022-21667-6 (PMC9633816; doi:10.1038/s41598-022-21667-6)
Supplement: Supplementary file 1 — Supplementary Figures. [file 41598_2022_21667_MOESM1_ESM.pdf]

P2X4 receptor stimulation enhances MrgprB2-mediated mast cell activation and pseudoallergic reactions in mice

Kazuki Yoshida[1]*, Shota Tanihara[1], Yuki Miyashita[1], Kosuke Obayashi[1], Masa-aki Ito[1], Kimiko Yamamoto[2], Toshiyashu Imai[3], and Isao Matsuoka[1]

[1] Laboratory of Pharmacology, Faculty of Pharmacy, Takasaki University of Health and Welfare, Takasaki-shi, Gunma 370-0033, Japan

[2] Department of Biomedical Engineering, Graduate School of Medicine, The University of Tokyo, Tokyo 113-0033, Japan

[3] Discovery Research Laboratories, Nippon Chemiphar Co., Ltd., Misato, Saitama 341-0005, Japan

* Corresponding author: yoshida-k@takasaki-u.ac.jp

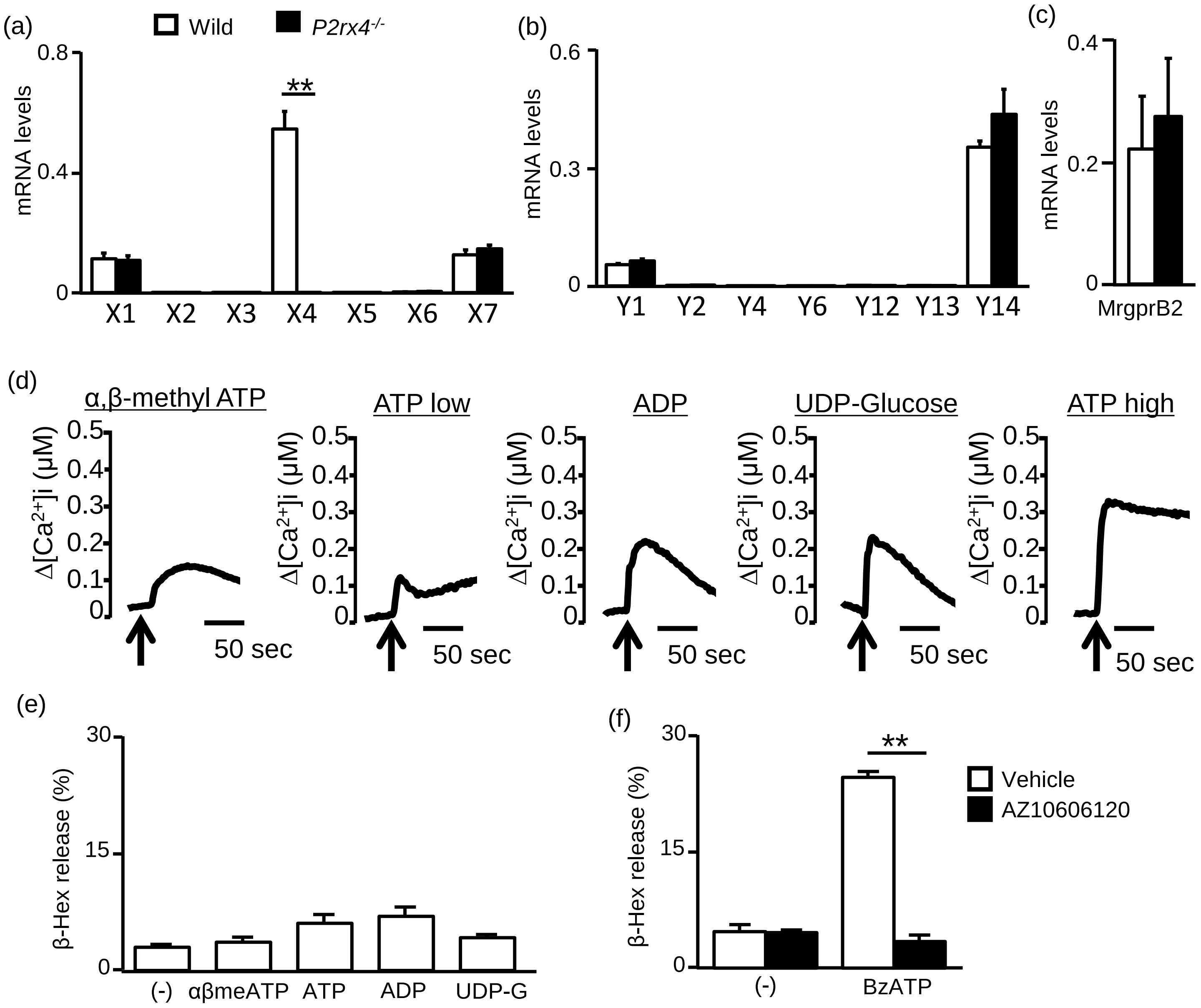

Supplementary Figure S1. Expression of P2 receptors mRNA and effect of purinergic receptor agonist on intracellular Ca^{2+} concentration ($[Ca^{2+}]i$) levels and degranulation in PMCs. P2X (a), P2Y (b) receptor and MrgprB2 (c) mRNA expression were analyzed by quantitative real time-PCR. mRNA levels were normalized by GAPDH (n=4). (d) PMCs preloaded with fura 2-AM were stimulated with P2X1 receptor agonist αβmeATP (10 μM), P2X receptor agonist ATP (100 μM), $P2Y_1$ receptor agonist ADP (100 μM), $P2Y_{14}$ receptor agonist UDP-G (100 μM) or 1 mM ATP (ATP high). Traces of typical changes in $[Ca^{2+}]i$ were shown as representative of 3-4 experiments. (e) PMCs were stimulated with αβmeATP (10 μM), ATP (100 μM), ADP (100 μM) and UDP-G (100 μM) for 10 min, and β-hexosaminidase (β-Hex) released into the reaction medium were measured (n=3). (f) PMCs were stimulated with P2X7 receptor agonist BzATP (300 μM) in the presence or absence of P2X7 receptor antagonist AZ10606120 (10 μM) (n=3). Data are presented as the mean ± SEM. **P<0.01

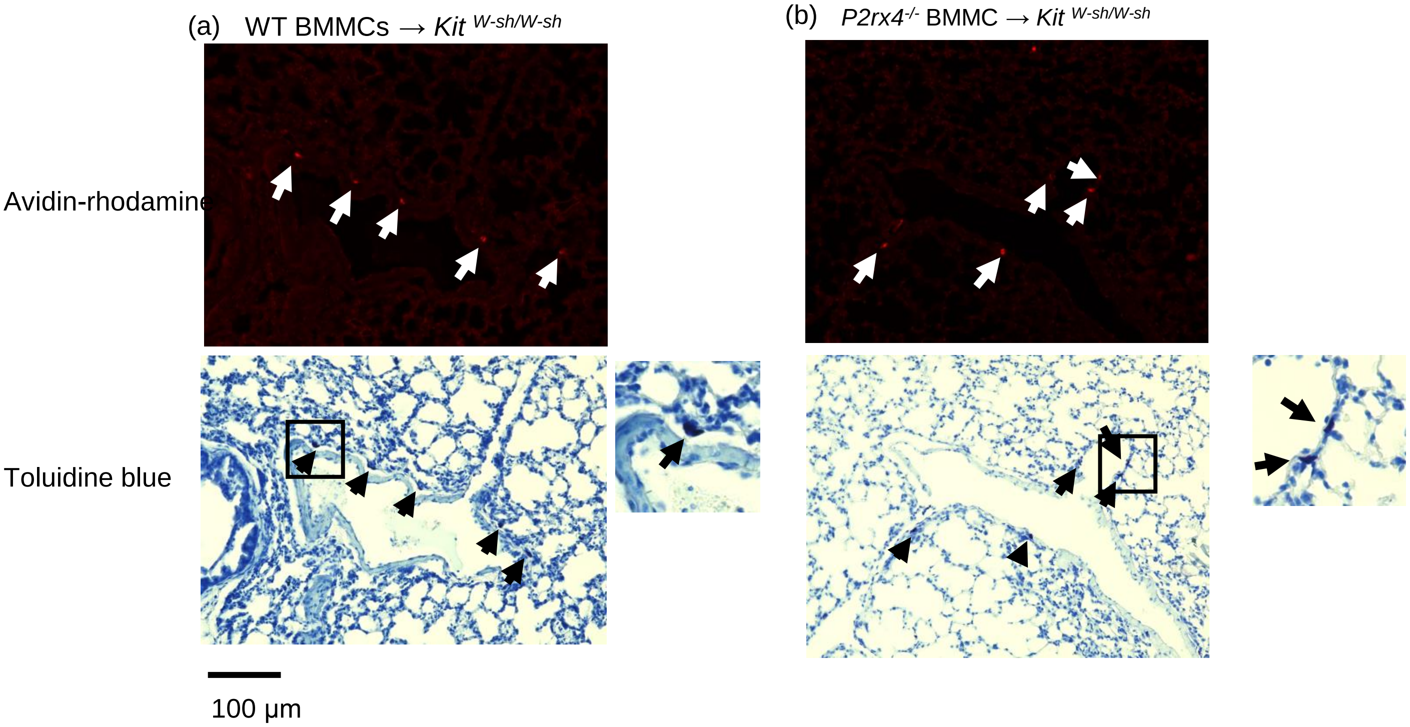

Supplementary Figure S2. Tissue distribution of reconstituted MCs in *Kit*$^{W\text{-}sh/W\text{-}sh}$ mice. MC deficient *Kit*$^{W\text{-}sh/W\text{-}sh}$ mice were intravenously injected with BMMCs prepared from WT (a) - and *P2rx4*$^{-/-}$ (b) mice. Four month later, lung sections were examined for reconstituted MC using avidin-rhodamine (upper panel) and toluidine blue stain (lower panel). Arrows indicate MCs.
